# The Effectiveness of Online Cognitive Behavioral Treatment in Routine Clinical Practice

**DOI:** 10.1371/journal.pone.0040089

**Published:** 2012-07-05

**Authors:** Jeroen Ruwaard, Alfred Lange, Bart Schrieken, Conor V. Dolan, Paul Emmelkamp

**Affiliations:** 1 Department of Clinical Psychology, University of Amsterdam, Amsterdam, The Netherlands; 2 Interapy PLC, Amsterdam, The Netherlands; 3 Department of Psychological Methods, University of Amsterdam, Amsterdam, The Netherlands; University of Western Brittany, France

## Abstract

**Context:**

Randomized controlled trails have identified online cognitive behavioral therapy as an efficacious intervention in the management of common mental health disorders.

**Objective:**

To assess the effectiveness of online CBT for different mental disorders in routine clinical practice.

**Design:**

An uncontrolled before-after study, with measurements at baseline, posttest, 6-week follow-up, and 1-year follow-up.

**Participants & Setting:**

1500 adult patients (female: 67%; mean age: 40 years) with a GP referral for psychotherapy were treated at a Dutch online mental health clinic for symptoms of depression (n = 413), panic disorder (n = 139), posttraumatic stress (n = 478), or burnout (n = 470).

**Interventions:**

Manualized, web-based, therapist-assisted CBT, of which the efficacy was previously demonstrated in a series of controlled trials. Standardized duration of treatment varied from 5 weeks (online CBT for Posttraumatic stress) to 16 weeks (online CBT for Depression).

**Main Outcome Measures:**

Validated self-report questionnaires of specific and general psychopathology, including the Beck Depression Inventory, the Impact of Event Scale, the Panic Disorder Severity Scale-Self Report, the Oldenburg Burnout Inventory, and the Depression Anxiety Stress Scales.

**Results:**

Treatment adherence was 71% (*n* = 1071). Study attrition was 21% at posttest, 33% at 6-week FU and 65% at 1-year FU. Mixed-model repeated measures regression identified large short-term reductions in all measures of primary symptoms (*d* = 1.9±0.2 to *d* = 1.2±0.2; *P*<.001), which sustained up to one year after treatment. At posttest, rates of reliable improvement and recovery were 71% and 52% in the completer sample (full sample: 55%/40%). Patient satisfaction was high.

**Conclusions:**

Results suggest that online therapist-assisted CBT may be as effective in routine practice as it is in clinical trials. Although pre-treatment withdrawal and long-term outcomes require further study, results warrant continued implementation of online CBT.

## Introduction

In the past decade, there has been a rapid expansion in the research and development of internet-based psychotherapeutic interventions. As a result, we now know that online interventions are feasible and efficacious in the prevention and treatment of a wide variety of common mental health disorders [1]–[4]. Although effect sizes vary with program characteristics (e.g., whether human support is included, or whether the aim is prevention or treatment), the benefits of various approaches are clear. Within the field, there is general agreement that online interventions are pivotal in improving the accessibility and uptake of evidence-based care [5].

While the benefits of online interventions have been firmly established in controlled research, the performance of these interventions in routine clinical practice is less clear [6]. Online interventions do not fit traditional healthcare systems, and raise legal, ethical, and professional issues that are only partially resolved by current guidelines [7]. Consequently, the implementation of online treatment in routine clinical practice has progressed slowly, which has limited the options for effectiveness research. Some interventions have been evaluated in real-world contexts, with positive results [8]–[15]. However, the current evidence base with regard to the effectiveness of online interventions in routine practice is small. Large-sample effectiveness studies are needed before wide-scale dissemination of online interventions can be recommended.

In this article, we present a study of the outcome of online therapist-assisted cognitive behavioral treatment (CBT) of 1500 patients, who were treated for symptoms of depression, panic disorder, posttraumatic stress, or burnout at a Dutch online mental health clinic. The efficacy of these treatments was previously demonstrated in seven randomized controlled trials, which included a total of 629 participants [16]–[24]. The objective of the present study was to assess the external validity of these trials, by examining the effectiveness of these treatments in routine clinical practice. Given the outcome of the controlled trials, we expected the treatment to produce large, significant, and clinically significant reductions in the relevant symptoms of psychopathology.

## Methods

### Study Design & Setting

This was an uncontrolled pre/post/follow-up study. Data were obtained from the electronic patients records of Interapy PLC, a Dutch online mental health clinic. These records provide data that are routinely collected before treatment, immediately after treatment, six weeks after treatment, and one year after treatment. In March 2009, we queried the electronic patient database of the clinic. Starting with the first record in the database (entry date: February, 2002), we retrieved consecutive records until we obtained data of *N* = 1500 patients, who had started treatment (entry date of last record: January, 2008).

### Participants

#### Patients

Patients were Dutch adults, who were screened through a series of validated web-administered self-report questionnaires and a 30-minute semi-structured diagnostic telephone interview. The clinic did not accept applicants, who a) showed signs of heightened risk of dissociation, psychosis, suicidal ideation, alcohol or drug dependence, b) were recently hospitalized because of mental health problems, c) used neuroleptic medication, d) used unstable doses of other psychoactive medication, or e) suffered from a prevailing disorder for which the clinic could not provide treatment. As a final requirement, the clinic demanded that every patient was seen by a General Practitioner (GP) or another health professional. The screening procedure was open to all, at no costs. However, treatment did not start without a (confirmed) referral source. Referrers received electronic reports at intake, halfway during treatment, and at posttest. Since this was a routine practice service evaluation, study approval was not obtained from an ethics committee. All patients approved the use of anonymized data through signed informed consent.

#### Therapists

Therapists were employed by or managed by the clinic. All had a university master’s degree in clinical psychology, completed extensive training in CBT, and received additional training in delivering the specific treatment manuals. Most therapists were junior therapists, who were employed by the clinic immediately after their graduation. They were supervised by two licensed clinical psychologists. Psychiatric consultation was available when needed.

### Interventions

All patients received web-based therapist-assisted CBT. Depending on presenting problems, patients were assigned to one of four manualized treatments for symptoms of depression, panic disorder, posttraumatic stress, or burnout. The treatment manuals were identical to those tested in previous controlled trials [16]–[24]. In these treatments, screening, treatment, and outcome measurement are conducted without any face to face contact. With exception of a diagnostic telephone interview, patients and therapists interact through a secure website, in the form of asynchronous text-messages (i.e., their dialogue resembles a structured e-mail conversation rather than a video-conference or an online chat-session).The manuals define fixed sequences of homework assignments that implement common CBT interventions, which are translated into a format suitable for delivery over the Internet. Therapist support consists of standardized, default feedback and instructions that are tailored by the therapist to the specific situation of the patient. In the feedback, motivational techniques are used to enhance the impact of the interventions, i.e., to ensure patients understand the purpose of the interventions, that they set realistic goals, that they do the exercises as prescribed, and that they continue treatment. Standardized duration and hours of therapist input of the treatments varies from 5 weeks and 9.5 hours (*Web-CBT for Posttraumatic Stress*) to 16 weeks and 19.5 hours (*Web-CBT for Depression*).

*Web-CBT for Depression* is a 16-week treatment that includes symptom awareness training, structuring of daily activities, challenging of dysfunctional thinking patterns, positive self-verbalization, social skills training and relapse prevention [21]. *Web-CBT for Panic Disorder* comprises 11 weeks of CBT, and includes symptom awareness training, applied relaxation, *in vitro* exposure, cognitive restructuring, *in vivo* exposure and relapse prevention [22]. *Web-CBT for Posttraumatic Stress* takes 5 weeks, and consists of structured writing exercises that implement imaginary exposure, cognitive reappraisal and social sharing [25]. *Web-CBT for Burnout* consists of 16-weeks of online CBT [20]. It comprises symptom awareness training, progressive relaxation, social skills training, positive self-verbalization [26], a rumination intervention, cognitive restructuring, time management training and relapse prevention.

### Measures

Patients received automated e-mailed invitations to complete a set of validated self-report questionnaires on the website of the clinic, at pretest, posttest, 6-week follow-up and at 1-year follow-up.

#### Primary outcomes

Primary outcomes were pre- to post-treatment changes in the intensity of specific psychopathology (i.e., symptoms of depression, panic disorder, posttraumatic stress, or burnout), as measured through validated questionnaires that were different for each treatment manual. *Web-CBT for depression* included the Beck Depression Inventory (BDI-IA; 22 items; range 0–65 [27], [28]), and the Depression subscale of the Depression Anxiety Stress Scales (14 items; range: 0–42 [29], [30]). *Web-CBT for panic disorder* included the Panic Disorder Severity Scale Self-report (PDSS-SR; 7 items; range: 0–45 [31], [32]). *Web-CBT for posttraumatic stress* included the Impact of Event Scale (IES; 15 items; range 0–75 [33], [34]). The assessment protocol of the treatment manual for posttraumatic stress did not include a one-year follow-up. *Web-CBT for burnout* included the Oldenburg Burnout Inventory (OLBI; 16 items, range of mean global score: 1–4 [35], [36]), and the Stress subscale of the DASS (14 items; range 0–42). These primary symptom measures were identical to those used in the clinical trials. Full descriptions of the questionnaires, which are often used in mental health outcome research, can be found in the reports of these trials [16]–[24]. The psychometric characteristics of these questionnaires are satisfactory (PDSS-SR, OLBI) to good (DASS, BDI, IES). In the present sample, Cronbach’s alpha’s ranged from α = .73 (OLBI) to α = .95 (DASS Depression).

#### Secondary outcomes

Secondary outcomes were pre-to-post-treatment changes in general psychopathology, and patient satisfaction. General psychopathology was assessed through the total score of the DASS (42 items; range: 0–126), which provides a global measure of negative emotional symptoms [29]. In the present sample, the internal consistency of this measure was excellent (Cronbach’s alpha: α = .97). Patient satisfaction was assessed through a brief evaluation questionnaire, which was administered at posttest. Patients rated the contact with their therapists (on a 1–10 scale), and stated whether they perceived the treatment as effective, whether they had missed the face to face contact, and whether they would recommend the treatment to others (*Yes/No/Don’t know*).

### Statistical Analyses

#### Statistical significance and effect size

Treatment effects were estimated through multi-level mixed-model repeated measures regression (MMRM) [37], using the statistical software package R [38]. Time of measurement was coded to contrast mean baseline scores to a) mean scores at the short-term post-treatment assessments (i.e., the post-test and the 6-week follow-up), and b) mean scores at one-year follow-up. Separate analyses were conducted on data from each treatment and each outcome measure, using two-sided tests and Bonferroni corrections to ensure a family-wise significance level of α = .05. We fitted three-level regression models, with repeated measurements nested in patients at level 1, patients nested in therapists at level 2, and therapists at level 3 [39]. Conditional intraclass correlations (ICC [40]) revealed that 2% of the variance in outcome was attributable to differences between therapists (mean ICC = .02; range: .001–0.13). To express effect sizes as Cohen’s *d* [41], fixed effect regression estimates and associated 95% confidence intervals were divided by pretest standard deviations.

#### Clinical significance

Following principles set out by Jacobson and Truax [42], pre- to post-treatment change scores of each patient were classified as follows: a) deterioration (change was negative and statistically reliable, i.e., it exceeded 1.96 times the standard error of the difference, b) no change (change was not statistically reliable), c) improvement (change was positive and statistically reliable), or d) recovery (change was positive, statistically reliable, and involved a change from a score above clinical cut-off to a score below this cut-off). Analyses were conducted on observed data of the full sample (i.e., *N* = 1500, assuming no change in the patients, who did not complete post-test measurements) as well as the completer sample (i.e., those patients, who completed the full treatment and post-treatment assessment).

## Results

### Sample Selection

**Figure 1:**
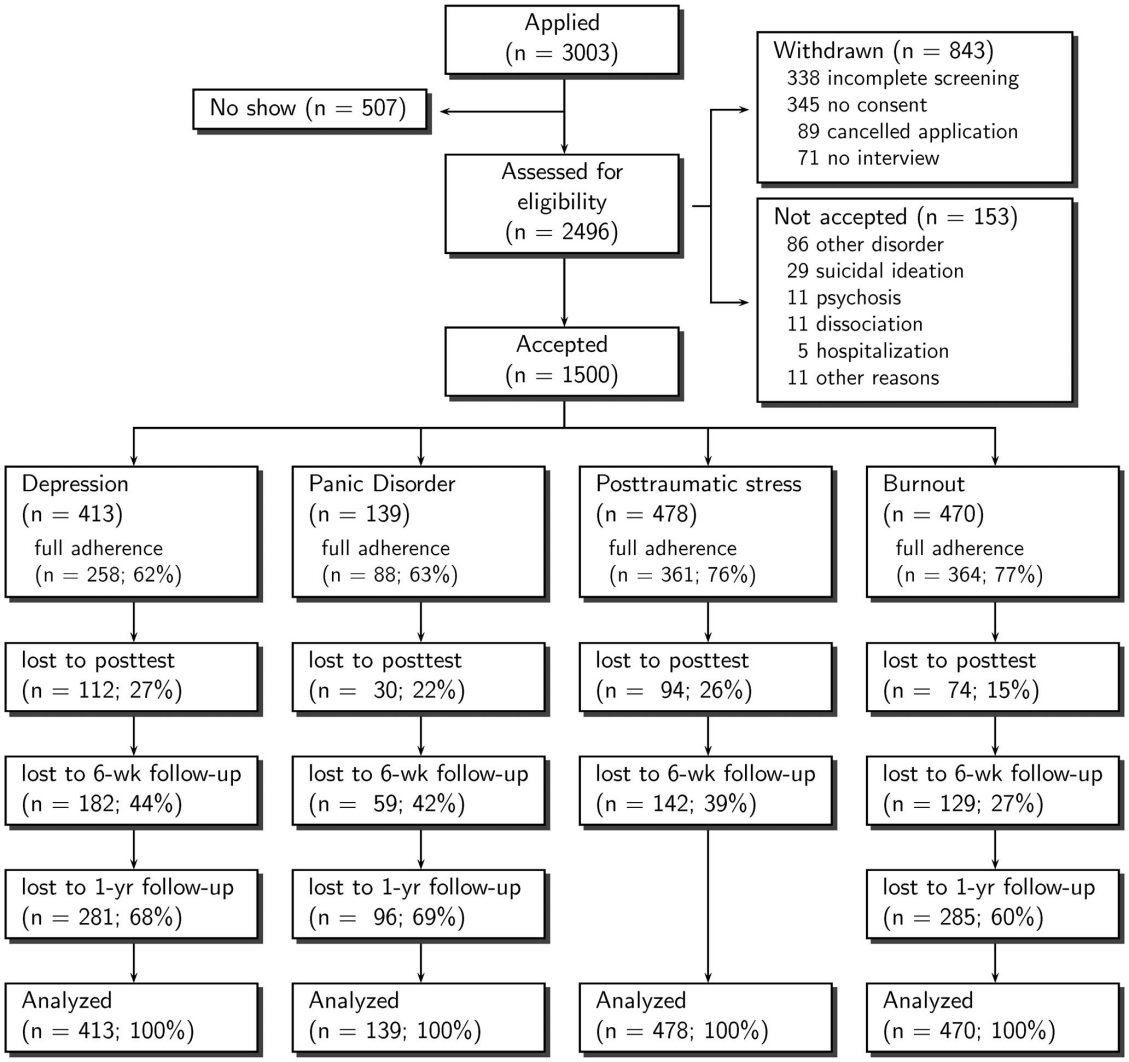
Patient flow.

To obtain 1500 records of patients who started treatment, 3003 patient records were retrieved from the database. Figure 1 shows that 507 (17%) of the applicants did not start baseline assessment, 843 (28%) withdrew during assessment, 153 (5%) were referred to other mental health institutions, while 50% (*N* = 1500) started treatment. The available data did not allow for a systematic analysis of the reasons of voluntary pre-treatment withdrawal. Of the 1500 accepted patients, 413 (28%) started *Web-CBT for depression*, 139 (9%) started *Web-CBT for panic disorder*, 478 (32%) started *Web-CBT for posttraumatic stress,* and 470 (31%) started *Web-CBT for burnout*. Patients were treated by a total of 135 therapists (depression: *n* = 74; panic disorder: *n* = 24; posttraumatic stress: *n* = 65; burnout: *n* = 51).

### Baseline Characteristics

Table 1 shows baseline characteristics of the patients in the study sample (*N* = 1500). The sample comprised 1011 women (67%) and 489 men (33%), who were between 14 and 73 years old (*M* = 40; *SD* = 11; only two patients were younger than 16). Most (*n* = 1052; 71%) did not use psychiatric medication. The vast majority of patients scored above clinical cut-off on the primary outcome measures (*n* = 1407, 94%; range 73%–99% across treatments). Patients were referred by GP’s (51%), other specialized mental health organizations (38%), or occupational health officers (11%).

**Table 1:** Baseline characteristics of patients.

Characteristic^a^		Depression	Panic Disorder	Posttraumatic Stress	Burnout
Female		280 (68%)	86 (62%)	369 (77%)	276 (59%)
Age M (SD)		40 (11)	37 (11)	38 (12)	41 (9)
Education	Low (secondary or less)	201 (49%)	73 (54%)	284 (61%)	198 (43%)
	High (tertiary or more)	207 (51%)	63 (46%)	182 (39%)	261 (57%)
Computer skills (1–10) M (SD)		7.8 (1.9)	8.1 (1.8)	7.6 (1.9)	6.9 (2.5)
Primary symtoms > clinical cut-off^b^		399 (97%)	101 (73%)	458 (96%)	449 (96%)
Years with symptoms	less than 1 year	187 (45%)	39 (28%)	231 (49%)	286 (61%)
	1 to 4 years	114 (28%)	40 (29%)	145 (31%)	141 (30%)
	5 years or more	110 (27%)	58 (42%)	96 (20%)	40 (9%)
Medication	no medication	237 (59%)	68 (50%)	362 (76%)	385 (83%)
	antidepressant	115 (28%)	21 (15%)	53 (11%)	39 (8%)
	anxiolytic	23 (6%)	29 (21%)	36 (8%)	31 (7%)
	combination	29 (7%)	19 (14%)	24 (5%)	8 (2%)

a Values represent subsamples and percentages unless otherwise noted. Counts do not add up to 1500 for every characteristic due to missing values (less than 5% of the data). Depression n = 413; Panic Disorder: n = 139; Posttraumatic Stress: n = 478; Burnout: n = 470.

b As measured through the global score of the Oldenburg Burnout Inventory (burnout; clinical cut-off: 2.18), the Beck Depression Inventory-IA (depression; cut-off: 10), the Panic Disorder Symptom Severity Self-Report (panic disorder; cut-off: 8) and the global score of the Impact of Event Scale (posttraumatic stress; cut-off: 24).

### Treatment Adherence, Treatment Duration, and Study Attrition

Treatment adherence, defined as the percentage of patients completing every step of the treatment program, was 71% (*n* = 1071). As shown in Figure 1, adherence was highest in the posttraumatic stress sample (76%) and the burnout sample (77%), and lowest in the depression sample (62%) and the panic disorder sample (63%). Patients completed an average of 84% of the treatment protocol, ranging from 81% (*Web-CBT for depression*) to 87% (*Web-CBT for burnout*). Treatments took a median of 22 weeks with depression (IQR = 12), 19 weeks with panic disorder (IQR = 11), 8 weeks with posttraumatic stress (IQR = 6), and 20 weeks with burnout (IQR = 13). Study attrition, defined as the percentage of patients not completing post-treatment measurements, was 21% at post-test (310/1500), 34% at 6-week follow-up (512/1500), and 65% at one-year follow-up (662/1022).

### Statistical Significance and Effect Size

#### Statistical significance

Table 2 shows means and standard deviations of patients’ questionnaire scores at each measurement occasion. Results of the regression analyses of pre-to-post-treatment changes in symptom severity are shown in Table 3. Each measure of specific and general psychopathology indicated significant (*P*<.001) reductions in symptom severity, on the short term (at posttest and 6-week follow-up), as well as on the long term (at one-year follow-up).

**Table 2:** Means and SDs of measures of specific and general psychopathology.

		Pretest		Posttest		6 Wk Follow-up		1 YR Follow-up	
Treatment	Measure^a^	n^b^	mean (SD)	n	mean (SD)	n	mean (SD)	n	mean (SD)
**Specific psychopathology**									
Depression	BDI	405	24.2 (8.0)	301	8.4 (7.7)	249	8.7 (7.7)	131	9.0 (7.6)
	DASS Depression		22.0 (9.1)		6.8 (8.1)		7.5 (7.8)		7.7 (8.1)
Panic disorder	PDSS-SR	136	11.5 (5.8)	109	4.5 (4.2)	80	4.8 (4.2)	43	3.1 (4.3)
Posttraumatic stress	IES Intrusion	477	24.6 (7.3)	384	13.0 (9.3)	336	11.7 (9.6)	c	
	IES Avoidance		23.4 (8.5)		12.0 (8.9)		11.2 (10.0)		
Burnout	OLBI Exhaustion	470	2.7 (0.6)	396	2.3 (0.6)	341	2.3 (0.6)	184	2.2 (0.7)
	OLBI Disengagement		3.0 (0.4)		2.3 (0.5)		2.3 (0.5)		2.2 (0.6)
	DASS Stress		19.7 (8.8)		7.7 (7.0)		7.9 (7.1)		7.3 (7.9)
**General Psychopathology**									
Depression	DASS global score	413	53.6 (21.1)	301	20.0 (19.6)	249	21.1 (19.5)	131	20.4 (18.7)
Panic disorder		136	47.3 (24.6)	108	20.2 (22.5)	80	20.0 (20.6)	44	15.2 (17.2)
Posttraumatic stress		476	43.5 (25.7)	381	24.2 (24.9)	334	23.6 (27.1)	c	
Burnout		470	44.2 (22.6)	399	15.8 (16.5)	342	16.1 (16.0)	185	14.8 (17.2)

a BDI: Beck Depression Inventory, version IA (range: 0–63; cut-off: 10); DASS: Depression Anxiety Stress Scales (DASS-42; range: 0–126; cut-off: 30, DASS-Stress: cut-off: 14). PDSS-SR: Panic Disorder Severity Scale - Self report (range: 0–28; cut-off: 8); IES: Impact of Event Scale (Intrusion subscale: range: 0–35; Avoidance Subscale: range 0–40; sumscore cut-off: 24 ); OLBI: Oldenburg Burnout Inventory (OLBI: range 1–4; cut-off: 2.18).

b Pretest data were missing for 8 patients in the depression sample, 3 patients in the panic sample, and 1 patient in the PTS sample.

c The assessment protocol of the treatment manual for posttraumatic stress did not include a one-year follow-up.

#### Effect size

Point estimates and 95% confidence intervals of effect sizes are shown in Table 3. With regard to the primary outcome measures of specific symptom severity, short-term improvements represented a large pooled standardized effect size of *d* = 1.4 (range: 0.7≤ *d* ≤1.9). One year after treatment, these effect sizes were found to be sustained. With regard to general psychopathology (as measured by the DASS total score), short-term improvements represented a large pooled effect size of 1.2 (range: 0.7≤*d*≤1.6), and these effect sizes also sustained on the long term.

**Table 3:** Regression analysis and effect sizes of changes in specific and general psychopathology.

			Baseline vs. Posttest/6 WK Follow-up			Baseline vs. 1 YR Follow-up		
Treatment		Measure^a^	b (SE) b	t^c^	*d* (CI_95_)^d^	b (SE)^b^	t^c^	*d* (CI_95_)^d^
**Specific psychopathology**								
Depression	BDI		−15.3 (0.46)	33.4	1.9 (±0.1)	−14.2 (0.82)	17.2	1.8 (±0.2)
	DASS Depression		−14.8 (0.52)	28.4	1.6 (±0.1)	−13.9 (0.74)	18.8	1.5 (±0.2)
Panic disorder	PDSS-SR		−6.6 (0.45)	14.6	1.2 (±0.2)	−7.5 (0.68)	11.1	1.3 (±0.3)
Posttraumatic stress	IES Intrusion		−12.0 (0.42)	28.3	1.6 (±0.1)			
	IES Avoidance		−11.4 (0.58)	19.6	1.3 (±0.1)	e		
Burnout	OLBI Exhaustion		−0.4 (0.03)	12.7	0.7 (±0.1)	−0.5 (0.05)	9.5	0.8 (±0.2)
	OLBI Disengagement		−0.7 (0.03)	23.6	1.6 (±0.1)	−0.8 (0.04)	18.5	1.8 (±0.2)
	DASS Stress		−11.9 (0.42)	28.1	1.3 (±0.1)	−12.1 (0.62)	19.4	1.4 (±0.1)
**General Psychopathology**								
Depression	DASS global score		−32.9 (1.23)	26.8	1.6 (±0.1)	−31.6 (1.70)	18.2	1.5 (±0.2)
Panic disorder			−26.1 (2.20)	11.9	1.3 (±0.2)	−29.7 (3.10)	9.7	1.2 (±0.3)
Posttraumatic stress			−18.9 (1.20)	15.5	0.7 (±0.1)	e		
Burnout			−28.2 (1.05)	27.4	1.2 (±0.1)	−28.7 (1.47)	19.6	1.3 (±0.1)

a BDI: Beck Depression Inventory, version IA; PDSS-SR: Panic Disorder Severity Scale - Self report; IES: Impact of Event Scale; OLBI: Oldenburg Burnout Inventory (OLBI); DASS: Depression Anxiety Stress Scales (DASS-42).

b b (SE): regression estimate and standard error of raw change score; Negative values of b represent symptom reductions.

c t: test statistic. N: depression: n = 413; panic disorder: n = 139; posttraumatic stress: n = 478; burnout: n = 470. Degrees of freedom of the t-test were conservatively set to the number of therapists in each sample (depression: n = 74; panic disorder: n = 24; posttraumatic stress: n = 65; burnout: n = 51). All regression parameters are significant at *P*<.001 after Bonferroni corrections for multiple testing.

d *d* (CI_95_): Cohen’s *d* effect size and 95% confidence interval.

e The assessment protocol of the treatment manual for posttraumatic stress did not include a one-year follow-up.

### Clinical Significance

Results of the clinical significance analyses are shown in Table 4. On the short-term (at post-test and 6-weeks follow-up), reliable improvement was 72% among treatment completers (*n* = 1046, 71%), and 55% in the full intent-to-treat sample (*N* = 1500, conservatively assuming no change where scores were unavailable). Recovery (i.e., reliable improvement from a pretest score above cut-off to a post-treatment score below cut-off) was 51% in the completers analysis and 40% in the intent-to-treat analysis. Reliable deterioration was 2% or less. Available data of the 1-year follow-up (n = 358), which were almost exclusively provided by treatment completers (99%), revealed an average reliable improvement rate of 78% and a recovery rate of 59% (c.f. Table 5).

### Patient Satisfaction

Posttest evaluation data, which were available for *n* = 1107 patients, are summarized in Table 6. Patient satisfaction was high, with little variance between treatment samples. Patients gave high ratings to their therapists (*M* = 8.5 on a 1–10 scale; *SD* = 1.5). Although 30% (*n* = 330) of the patients indicated that they had missed face-to-face contact during therapy, 83% evaluated online therapy as effective, and 89% would recommend web-based treatment to others.

## Discussion

### Key Findings

We assessed the effectiveness of online CBT in routine clinical practice among 1500 patients, who suffered from symptoms of depression, panic disorder, posttraumatic stress, or burnout. We found that effect sizes and recovery rates were comparable to, or somewhat better than, those observed in previous controlled trials [16]–[22], and comparable to those of face-to-face routine practice CBT [43]–[46]. Post-treatment reductions of specific and general psychopathology were large (pooled *d* = 1.4/1.2; *P*<.001), about 50% of the patients recovered, and patient satisfaction was high. Our findings suggest that online CBT may be as effective in routine practice as it is in clinical trials.

While high dropout is a common problem in studies of online interventions [47]–[50], our data show that acceptable adherence can be achieved with online treatment. We found that 79% of the patients completed post-treatment measurements, and that 71% completed every step of treatment. Although adherence rates were somewhat better in the controlled trials (83%), these findings still compare well to published adherence rates of Dutch routine practice mental healthcare, which tend to vary between 60% to 70% [51].

As previously shown by Titov, Andrews, Kemp, and Robinson, patients who seek online treatment have substantial disorders, and are not necessarily young or technologically sophisticated [52]. Our data confirm and extend this finding. In terms of demographic characteristics and presenting problems, the patients of the online clinic are comparable to the patients that are generally seen in Dutch specialized mental healthcare. Our results indicate that online treatment provides an appropriate intervention for these patients.

### Strengths & Limitations

This study has several strengths. First, the treatments had been carried out before the study was planned. Treatment outcomes were routinely assessed as part of every-day practice. Accordingly, therapists and staff were not influenced by their participation in the evaluation study (the so-called Hawthorne effect [53]). Second, the size of the sample is much larger than the sample sizes of previous effectiveness studies of therapist-assisted online CBT. Third, the sample included every patient who had started treatment in the studied timeframe. Thus, our results could not be affected by a selection of well-responding patients. Fourth, patients were treated by a large number of relatively inexperienced therapists. Since we found little variance between the therapists in terms of treatment outcome, we concur with Wilson, who argued that “the capacity to train a diverse group of therapists to a criterion level of competence so that they can reliably administer a treatment protocol […] can be seen as a significant advance in the dissemination of effective treatment” [54]. Fifth, the therapeutic procedures and outcome measures in the study were identical to those used in the controlled trials. In both contexts, the same computerized treatment manuals were used. Hence, treatment integrity was guaranteed in both contexts. Sixth, there was no face-to-face contact at all. This considerably enhances the flexibility of the online treatment, since it provides the possibility to treat patients who live at distant locations. Paradoxically, this positive aspect could also be seen as a potential weakness, as will become clear in our subsequent discussion of the limitations of the study.

**Table 4:** Clinical significance of short-term^a^ changes in primary psychopathology.

Treatment	Measure^b^	Sample^c^	n	Recovered	Improved	No change	Deteriorated
Depression	BDI	All	413	182 (44%)	64 (15%)	164 (40%)	3 (1%)
		Completer	258	162 (63%)	55 (21%)	40 (16%)	1 (0%)
	DASS Depression	All	413	191 (46%)	59 (14%)	157 (38%)	6 (1%)
		Completer	258	168 (65%)	49 (19%)	36 (14%)	5 (2%)
Panic disorder	PDSS-SR	All	139	50 (36%)	15 (11%)	74 (53%)	0 (0%)
		Completer	87	40 (46%)	12 (14%)	35 (40%)	0 (0%)
							
Posttraumatic stress	IES	All	478	190 (40%)	94 (20%)	193 (40%)	1 (0%)
		Completer	358	181 (51%)	93 (26%)	83 (23%)	1 (0%)
							
Burnout	OLBI	All	470	134 (29%)	111 (24%)	220 (47%)	5 (1%)
		Completer	364	122 (34%)	104 (29%)	133 (37%)	5 (1%)
	DASS Stress	All	470	205 (44%)	58 (12%)	204 (43%)	3 (1%)
		Completer	364	193 (53%)	57 (16%)	111 (30%)	3 (1%)

a Analyses of short-term changes were based on individual difference scores on the primary outcome measures, calculated as the pretest score minus the mean of the post-test and 6-week follow-up score.

b BDI: Beck Depression Inventory, version IA (cut-off: 10; reliable change: 7 scale points); DASS: Depression Anxiety Stress Scales (DASS Depression cut-off: 12; reliable change:5 scale points; DASS Stress cut-off: 14; reliable change: 7 scale points); IES: the Impact of Event Scale (cut-off: 24; reliable change: 12 scale points); OLBI: Oldenburg Burnout Inventory (cut-off: 2.18; reliable change: 37 scale points). PDSS-SR: Panic Disorder Severity Scale - Self report (cut-off: 8; reliable change: 5 scale points).

c All: all patients (assuming no change where data was missing); Completer: subsample of patients, who completed the full treatment and at least one post-treatment assessment.

**Table 5:** Clinical significance of changes in primary psychopathology, at one-year follow-up.

Treatment	Measure^a^	n	Recovered	Improved	No change	Deteriorated
Depression	BDI	131	73 (56%)	33 (25%)	25 (19%)	0 (1%)
	DASS Depression		80 (70%)	19 (17%)	14 (12%)	2 (2%)
Panic disorder	PDSS-SR	43	28 (65%)	6 (14%)	9 (21%)	0 (0%)
Burnout	OLBI	184	72 (42%)	52 (30%)	40 (23%)	8 (5%)
	DASS Stress		95 (52%)	31 (17%)	54 (29%)	4 (2%)

a BDI: Beck Depression Inventory, version IA (cut-off: 10; reliable change: 7 scale points); DASS: Depression Anxiety Stress Scales (DASS Depression cut-off: 12; reliable change: 5 scale points; DASS Stress cut-off: 14; reliable change: 7 scale points); OLBI: Oldenburg Burnout Inventory (cut-off: 2.18; reliable change: 37 scale points). PDSS-SR: Panic Disorder Severity Scale - Self report (cut-off: 8; reliable change: 5 scale points).

**Table 6:** Patient satisfaction.

Aspect		Depression	Panic Disorder	Post-traumatic Stress	Burnout
Satisfaction with therapist M (SD)		8.4 (1.5)	8.7 (1.6)	8.6 (1.5)	8.6 (1.2)
Do you consider online therapy an effective method?	Yes	240 (81%)	90 (83%)	298 (78%)	289 (89%)
	No	30 (10%)	7 (6%)	29 (8%)	6 (2%)
	Don’t know	26 (9%)	11 (10%)	53 (14%)	28 (9%)
Did you miss face to face contact?	Yes	35 (33%)	35 (32%)	110 (29%)	87 (26%)
	No	163 (55%)	64 (59%)	231 (61%)	189 (59%)
	Don’t know	35 (12%)	9 (8%)	39 (10%)	47 (15%)
Would you recommend online treatment to others?	Yes	260 (88%)	95 (88%)	339 (89%)	292 (90%)
	No	18 (6%)	3 (3%)	18 (5%)	12 (4%)
	Don’t know	18 (6%)	10 (9%)	23 (6%)	19 (6%)

a Depression: n = 296; Panic Disorder: n = 108; Posttraumatic Stress: n = 380; Burnout: n = 323.

A first limitation of our study is that patient screening was conducted online and by telephone, without a structured clinical interview. The clinic made use of dimensional screening through validated self-report instruments for which norm tables have been established. This did not allow for formal DSM-IV diagnoses, but compared well with the DSM-IV categories by using the cut-off scores of the scales. Since there are indications that telephone interviews and face-to-face interviews yield comparable results [55], the clinic at present makes use of structured diagnostic interviews in telephone contacts during the screening, in addition to the dimensional screening. Second, we recall that a high percentage of the patients did not complete the long-term follow-up measures (65%). Although available data suggest that effects maintain up to one year after treatment, these results should be interpreted with caution. It would have been useful if we would have sent short questionnaires to non-responders, to assess whether there were any differences between responders and non-responders. Third, a considerable percentage of the applicants (40%) withdrew, during or even prior to the screening. While pre-treatment withdrawal rates are often not reported, research shows that high withdrawal rates are common, in both online and offline treatment [56], [57]. It is a challenge to improve these figures, but our data do not permit valid assessment of the reasons for and effects of pre-treatment withdrawal. The withdrawal might be caused by the ease with which one can apply for online therapy. This may result in impulsive applications (we found some indications that applicants, who started the screening without a referral, were less likely to start treatment). A second possibility is that patients withdraw from online treatment because they are unwilling to relinquish their anonymity [58].

### Clinical Utility

The protocols that are used in this routine practice comprise a strong mix of cognitive behavioral procedures and techniques that enhance patient motivation and the patient-therapist relation. One particular advantage of the therapy format is that therapists do not have to respond immediately when patients post their homework. Due to the asynchronous communication, therapists have time to reflect on the best possible feedback and the best explanation of new homework assignments. If needed, they may even discuss a case with colleagues. In addition, the Interapy protocols provide help files, which the therapists may consult to find motivating phrases for their feedback. All these aspects make this a highly usable treatment for clinical practice.

The study shows how computer-mediated treatment can save time by freeing therapists of repetitive tasks such as the administration and scoring of outcome questionnaires. In a computerized environment, these tasks can be easily automated.

Online treatment is highly dependent on regulatory approval, professional codes of ethics and jurisdiction regulations, which vary considerably from country to country. But even when such barriers have been overcome, financial hurdles may still exist. When the Interapy clinic was founded, in 2001, costs of face-to-face treatment were fully reimbursed by Dutch public health insurance, while costs of online treatment were not. In effect, access to online treatment was limited due to a financial barrier. This changed in 2005, when health regulatory bodies recognized the online services of the clinic as reimbursable healthcare, under condition of a GP-referral for psychotherapy. Without this recognition, further implementation of online CBT would not have been feasible.

At present, cost-benefit comparisons between face-to face-treatment and online treatment are complicated by the limited availability of routine practice data and by unclear definitions of relevant health-economic variables. Nonetheless, scant research has shown that online treatment is cost-effective [12], [59], and it would be instructive to conduct a cost-effectiveness study for the present treatment as well. One important variable in this respect is the amount of therapist input that is required for a meaningful clinical response. Less input implies fewer costs, but may also limit the effects of the intervention [60], [61].

### Conclusion

In sum, our study suggests strongly that RCT findings of online therapist-assisted CBT generalize well to routine clinical practice as carried out in the Interapy clinic. It would be interesting to examine whether results with this form of online CBT are similarly positive in other clinical contexts. While pre-treatment withdrawal and long-term outcome demand attention, our results suggest that online CBT provides a viable, effective, and acceptable treatment alternative for patients, who are unwilling or unable to seek traditional forms of mental healthcare. Future studies should confirm this suggestion through direct comparisons of online CBT and regular treatment options, preferably in the form of large-sample equivalence trials that are conducted in naturalistic settings. Meanwhile, based on our present results, we recommend further implementation of online CBT.
